# Notes of Surgical Cases Treated at the Buffalo General Hospital

**Published:** 1870-06

**Authors:** D. W. Harrington

**Affiliations:** Acting Resident Physician


ART. II.—*Notes of Surgical Cases Treated at the Buffalo General Hospital. Reported by* D. W. HARRINGTON, *Acting Resident Physician.*

Horace Dawney, aged 25, admitted to the hospital April 12th, with a discharging ulcer on each side of thigh, four inches above knee joint. They were supposed to be caused by an injury received eight years before while wrestling. There was also partial anchylosis of knee joint. April 18th, Dr. Gay operated by making an opening about four inches long on outer side of leg, and removing a large amount of necrosed bone. The dressing used was a weak solution of carbolic acid. May 19th, the patient was up and dressed; the wound is fast closing, with every prospect of finally obtaining very good result.

Charles Wanless, aged 11, admitted into hospital April 13th, with double congenital scrotal hernia. The external abdominal ring was large enough to admit two fingers, and when the intestines were down the scrotum was larger than a "man's fist." April 29th, Dr. Gay operated on one side by passing silver wire to connect pillars of external ring, making points of entry and exit of wire through cutaneous surface, about nine lines apart. A silk seaton was also passed just below the wire, to increase inflammation, which was left in fourteen days. May 1st, the wires cut the integument between points of entry and exit; it was then tightened, bringing into closer apposition and nearly closing the ring. May 18th, the ends of the twisted wire were cut off; the opening made by the wire in cutting through the integument was united by straps that it might heal over the wire which was left. There was very little constitutional disturbance, and no signs of peritonitis. The pulse was never over 100. May 21st, the boy was up and dressed, and it can safely be called a "radical cure."

Daniel Conners, aged 26, was admitted into hospital February 24th, suffering from gonorrhœa of eighteen months standing; also a stricture situated in membranous portion of urethra. He was a patient at the same hospital during the months of August and September of the preceding year, and was treated by passing bougie, or sound, (the largest used being No. 10,) and allowing it to remain as long as patient could bear it; also by the use of different injections and general tonic treatment, with abstinence from fats, condiments, and spirits. He left the hospital September 30th, partly relieved from the stricture, but the discharge was about the same. During the fall and early part of winter he was under the treatment of Dr. Chimes, of New York City, and was a short time in Bellevue hospital, but with no improvement. He was readmitted into this hospital February 24th. The stricture was treated as before with good result. No. 12 sound could be passed without difficulty, yet the discharge continued. April 29th, Dr. Gay passed Lallemand's porte caustique, and the urethra was cauterized at the original seat of stricture, and a short distance external to it. This was repeated after four days. He was given a f℥i every four hours of the mist.

Ant. et Pot. Tar^t., - - - - - - gr. ii,

Morphiæ Sulphat, - - - - - gr. ii,

Aquæ, - - - - - - - - ℥ii.

May 21st. The stricture has entirely disappeared, and there has been no discharge for two weeks.

